# Phage-Selected Clickable Gln-Donor Peptide for Lys-Selective Fab Labeling Using Engineered Microbial Transglutaminase

**DOI:** 10.3390/antib15040056

**Published:** 2026-06-26

**Authors:** Eva Agustriana, Koki Murozono, Kosuke Minamihata, Riko Nishioka, Noriho Kamiya

**Affiliations:** 1 Department of Applied Chemistry, Graduate School of Engineering, Kyushu University, 744 Motooka, Nishi-ku, Fukuoka 819-0395, Japan; agustriana.eva.012@s.kyushu-u.ac.jp (E.A.); murozono.koki.698@s.kyushu-u.ac.jp (K.M.); nishioka.riko.313@s.kyushu-u.ac.jp (R.N.); 2 Division of Biotechnology, Center for Future Chemistry, Kyushu University, 744 Motooka, Nishi-ku, Fukuoka 819-0395, Japan

**Keywords:** fusion protein, site-specific protein labeling, phage display, proximity labeling

## Abstract

**Background/Objectives**: The use of cross-linking enzymes for site-selective and efficient antibody modification has attracted considerable attention. Microbial transglutaminase (MTG)-mediated labeling of IgG at Gln295 has emerged as a promising strategy for preparing antibody–drug conjugates (ADCs). By contrast, selective modification of a specific Lys residue on native antibody surfaces using MTG remains challenging because most Lys residues exhibit low intrinsic reactivity. Here, we address this challenge by exploiting enzyme–antibody proximity together with screening for highly reactive Gln-donor substrates from a random peptide library. **Methods**: Reactive Gln-donor peptide substrates were first identified from a seven-amino-acid phage-displayed peptide library using a reactive Lys-containing peptide as bait. Based on the obtained sequence, an azide-functionalized Gln-donor peptide suitable for click chemistry was designed. **Results**: The designed substrate enabled efficient Lys65-selective modification of Fab fragments using a fusion of an engineered MTG zymogen and protein G (EzMTG-pG), followed by functionalization through click chemistry to yield fluorescent Fab conjugates. **Conclusions**: These results provide practical guidelines for substrate design in MTG-mediated site-selective protein modification.

## 1. Introduction

Site-selective modification of antibodies is an important strategy for expanding the functionality of antibody-based biomolecules. In particular, precise modification of Fab fragments has attracted considerable attention because they retain antigen-binding activity while offering advantages such as smaller molecular size and improved tissue penetration compared with whole antibody molecules [1,2]. These properties make Fab fragments attractive platforms for applications including molecular imaging probes, targeted therapeutics, and functional antibody conjugates [2,3,4,5,6].

Among various strategies for site-specific antibody modification, enzymatic approaches have gained increasing attention because they enable highly selective reactions under mild conditions. Several enzyme-mediated systems have been developed for antibody or Fab modification. For example, lipoic acid ligase has been used to modify engineered antibody fragments site-specifically [7], whereas sortase-mediated ligation has been used to modify IgG antibodies by exploiting proximity effects [8]. In these enzymatic protein modification systems, the design of suitable peptide substrates is crucial for determining reaction efficiency and selectivity.

Microbial transglutaminase (MTG) is a particularly attractive enzyme for antibody modification because it catalyzes cross-linking reactions between glutamine (Gln)-donor substrates and either primary amine-containing or lysine (Lys)-acceptor substrates. Early studies demonstrated that selective modification of IgG at Gln295 (Q295) can be achieved using MTG, by exploiting the broad tolerance of this enzyme for primary amine substrates. However, deglycosylation treatment was required to improve access to Q295 [9,10,11,12]. More recently, selective labeling of Q295 without deglycosylation has been achieved by designing peptide substrates containing highly basic Lys-acceptor peptidyl substrates [13]. These findings highlight the importance of substrate design in MTG-mediated antibody modification at a specific Gln residue.

Due to the limited number of Gln residues available for modification via MTG catalysis in antibodies, specifically Q295 [9], this residue has served as a reliable target for site-selective modification. However, since this Gln residue is located within the Fc region, this approach is not applicable to antibody formats lacking the Fc domain, such as antibody fragments. As an alternative target, Lys residues represent attractive reactive sites for antibody modification due to their abundance and solvent accessibility [14]. Although MTG accepts a wide range of primary amine substrates [15,16,17,18], most Lys residues on antibody surfaces are poorly recognized by the enzyme [19]. Nevertheless, recent advancements in site-selective Lys functionalization have been achieved. For instance, a previous study utilizing immobilized MTG demonstrated that Lys functionalization in aglycosylated IgG occurs site-specifically at the K340 position [20]. Moreover, K447 can be recognized by MTG, provided that a single C-terminal amino acid extension is present [19]. Despite these advancements in site-selective lysine modification by MTG, the functionalization of native Lys residues on antibodies without requiring prior genetic engineering or glycan removal remains highly desirable. Furthermore, although substrate selectivity for Gln-donor substrates is generally considered more stringent than that for primary amine substrates [21], various peptide sequences have been reported to function as effective Gln-donor substrates [21,22,23,24,25,26]. These observations suggest that molecular design considering both Lys-acceptor and Gln-donor substrates is essential for controlling MTG-catalyzed cross-linking reactions.

We recently reported that a fusion protein composed of an engineered zymogen of MTG (EzMTG) and protein G (EzMTG-pG) enables the modification of a specific Lys residue (K65) on the surface of a native IgG antibody (trastzumab) [27] or its Fab fragment [28] in a tag-free manner. In this system, the interaction between protein G and the antibody generates a proximity effect that enhances the modification of a Lys residue with intrinsically low EzMTG reactivity. Moreover, the selective modification of Lys residues on antibody surfaces using MTG requires the rational design of appropriate Gln-donor peptide substrates. In previous studies, we focused on the proximity effects using a Gln-donor substrate with a hydrophobic dye at the N-terminus, TAMRA-YPLQMRG-NH_2_, which served as a model drug payload. Under EzMTG-pG catalysis, highly efficient and site-selective modification of K65 in native IgG and Fab fragments was achieved [27,28]. However, the method used to identify such a reactive Gln-donor sequence has not yet been reported.

Based on these findings, the present study discloses the identification of short, MTG-reactive Gln-donor peptide sequences for the first time. Additionally, we present the design of reactive Gln-donor substrates for MTG-catalyzed labeling of specific Lys residues in a protein of interest, specifically, a Fab fragment, which is compatible with a broad range of biotechnological applications. To achieve this, first, potent Gln-donor peptide sequences were screened using a phage display system combined with a highly reactive Lys-containing peptide substrate as bait. Based on the identified sequences, azide-functionalized Gln-donor substrates suitable for click chemistry were designed and validated. Finally, their utility was evaluated through site-specific fluorescent labeling of Fab fragments.

## 2. Materials and Methods

### 2.1. Materials

Microbial transglutaminase (MTG) was kindly provided by Ajinomoto (Tokyo, Japan). All peptides were purchased through the custom peptide synthesis services. Biotin-GSMKHKGS was from GeneNet (Fukuoka, Japan), and azido-FYPLQMR-NH_2_, azido-FYPLQMRG-NH_2_, and azido-YPLQMRG-NH_2_ were from Sigma-Aldrich (St. Louis, MO, USA). DBCO-PEG4-TAMRA was purchased from BROADPHARM (San Diego, CA, USA). The Ph.D.-7 Phage Display Peptide Library and *Escherichia coli* ER2738 were obtained from New England Biolabs (Ipswich, MA, USA). Salmon sperm DNA was purchased from Bio Dynamics (Tokyo, Japan). Cystamine was obtained from Sigma-Aldrich (St. Louis, MO, USA). Siliconized microcentrifuge tubes were purchased from Biomedical Science Co. (Tokyo, Japan). All other reagents were of analytical grade and used as received. The recombinant expression and purification of EzMTG-pG, as well as the preparation of Fab from IgG antibody (trastuzumab), were conducted according to the protocol established in our previous study [28].

### 2.2. Phage Display Selection of MTG Substrate Sequences

The selection of preferred Gln-donor substrate peptide sequences for microbial transglutaminase (MTG) was performed using an M13 phage display random heptapeptide library (Ph.D.-7^TM ^ Phage Display Peptide Library) according to a previously reported procedure with modifications to the substrate molecules [21].

In the first round, phage particles (1 × 10^13^ pfu/mL, 10 µL) were incubated at 37 °C in the presence of biotin-GSMKHKGS peptide (final concentration, 0.5 mM) and MTG (0.33 ng/µL) in Tris-buffered saline (TBS, 10 mM Tris-HCl, 150 mM NaCl, pH 8.0) containing 5 mM DTT in a total volume of 100 µL. Reaction mixtures were preincubated at 37 °C for 5 min before MTG was added to initiate the reaction. MTG reactions were terminated by adding 110 mM cystamine (10 µL). From the second round onward, amplified phage suspension (40 µL) obtained from the previous round was used under the same reaction conditions.

Following the reaction, phage particles were precipitated by adding polyethylene glycol (PEG)–NaCl solution (final concentration: 3.3% PEG 6000 and 0.42 M NaCl) together with 10 ng of salmon sperm DNA as a carrier. After incubation on ice, precipitated phage particles were collected by centrifugation and resuspended in 400 µL of TBS buffer.

Biotinylated phage particles were captured using SoftLink Soft Release Avidin Resin (Promega, WI, USA). Briefly, 40 µL of 50% slurry of the avidin resin was added to the phage suspension and incubated for 1 h with gentle rotation. The resin was subsequently washed with TBSTE buffer (TBS containing 0.1% or 0.5% Tween-20 and 2 mM EDTA) followed by TBS buffer to remove unbound phage particles. Bound phage particles were eluted by incubation with 200 µL of TBS buffer containing 5 mM biotin. The elution step was performed twice, and a total of 400 µL of eluted phage solution was recovered.

Eluted phage particles were amplified by infection of exponentially growing *Escherichia coli* ER2738 cells. The phage solution was added to 20 mL of ER2738 culture, and the mixture was incubated at 37 °C for 4.5 h with aeration. Phage particles were subsequently recovered from the culture supernatant by PEG/NaCl precipitation (final concentration: 3.3% PEG 6000 and 0.42 M NaCl), followed by resuspension in 200 µL of TBS buffer. This phage suspension was used for the next round of selection.

We performed five rounds of panning. Selection stringency was progressively increased by shortening the MTG reaction time (15 min in the 1st round, 10 min in the 2nd round, and 1, 3, and 5 min in later rounds) and by increasing the Tween-20 concentration in the washing buffer (0.1% in the 1st round and 0.5% from the 2nd round onward). Phage titers were determined by a plaque assay using IPTG/X-gal indicator plates. Individual plaques from the final round were isolated and amplified, and single-stranded phage DNA was purified for sequencing. DNA sequences encoding the displayed peptides were determined by Sanger sequencing.

### 2.3. Enzymatic Labeling of Fab with Azide-Modified Gln-Donor Substrates by EzMTG-pG

To label Fab with an azide-modified Gln-donor, Fab (2.6 µM) was mixed with EzMTG-pG (2.6 µM) in 40 mM Tris-HCl (pH 8.0), the azide-modified Gln-donor substrates (100 µM) were added, and the reaction was carried out at 37 °C, for 2 h. Here, three types of azide-modified Gln-donor substrate were used: azido-FYPLQMR-NH_2_ (azido-acetyl-FYPLQMR-NH_2_), azido-FYPLQMRG-NH_2_ (azido-acetyl-FYPLQMRG-NH_2_), and azido-YPLQMRG-NH_2_ (azido-acetyl-YPLQMRG-NH_2_). After 2 h, the reaction was terminated by adding *N*-ethylmaleimide (NEM) at 1 mM. To evaluate the reactivity of the substrates, 30 µL of the reaction mixture was analyzed by RP-HPLC analysis under reducing condition using a Cosmosil 5Ph-AR-300 (4.6 mm ID × 150 mm) with detection at 214 nm, as previously reported [27,28]. To estimate the modification rate from using each substrate, the overlapping modified and unmodified heavy-chain peaks were deconvoluted using the Multipeak Fit in Igor Pro 9 (WaveMetrics, Inc., Lake Oswego, OR, USA), applying the exponentially modified Gaussian (ExpModGauss) function. The modification ratio was then calculated using Equation (1), where *A_mod_* and *A_unmod_* represent the integrated area of the fitted peaks for the modified and unmodified heavy chain, respectively.

(1) $$\mathrm{Modification}\;\mathrm{rate}\;\left(\%\right)\;=\frac{A_{mod}}{A_{mod}+A_{unmod}}\times 100$$

### 2.4. Evaluation of Binding Affinity of the Azide-Modified Fab

To investigate if the azide labeling by EzMTG-pG(Fab) catalysis alters the ability of Fab in binding with target antigen, the azide-modified Fab, obtained from the above method, was subjected to an antigen-binding affinity assay by BLItz (Fortebio, Fremont, CA, USA), which was conducted in duplicate (*N* = 2), following previously reported procedure [29].

### 2.5. Analysis of Modification Site in Fab

The modification position of azide-modified Gln-donor substrate in Fab was investigated by peptide mapping analysis conducted as reported previously [28,29].

### 2.6. Preparation of TAMRA-Modified Fab by Click Reaction

TAMRA-modified Fab was prepared by enzymatic conjugation of the azide functionality to the Fab, followed by a click reaction with TAMRA-PEG4-DBCO. In the first step, Fab (2.6 µM) was reacted with azide-modified Gln-donor (100 µM) under EzMTG-pG (2.6 µM) catalysis at 37 °C for 2 h. The reaction mixture was subsequently mixed with 0.1 M citrate buffer at pH 3, at a volume twice that of the reaction mixture, and injected onto a Protein Ark HiFliq S-type column (1 mL). The sample was purified using two buffer systems, 50 mM citrate with 0.2 M L-arginine hydrochloride, pH 3 (buffer A), and 50 mM citrate with 0.5 M L-arginine hydrochloride in 2 M NaCl, pH 3 (buffer B). After sample injection, elution was carried out by gradient of A/B from 95:5 to 45:55 over 20 min at 1.2 mL min^−1^. Fractions containing the conjugate, Fab–azide, were neutralized with 100 µL of 1 M Tris-HCl (pH 8), concentrated using an Amicon Ultra-15 device fitted with a 10,000 MWCO membrane (Merck, Darmstadt, Germany), and exchanged into PBS (pH 7.4). Subsequently, purified Fab–azide (10 µM) was reacted with TAMRA-PEG4-DBCO (100 µM) in PBS (pH 7.4) for 2 h. The excess of TAMRA-PEG4-DBCO was removed by passing through the reaction mixture into a PD-10 G-25 SpinTrap column. Sample was then analyzed by RP-HPLC and SDS-PAGE analysis.

## 3. Results

### 3.1. Screening of MTG Substrates Using Phage Display

**Figure 1:**
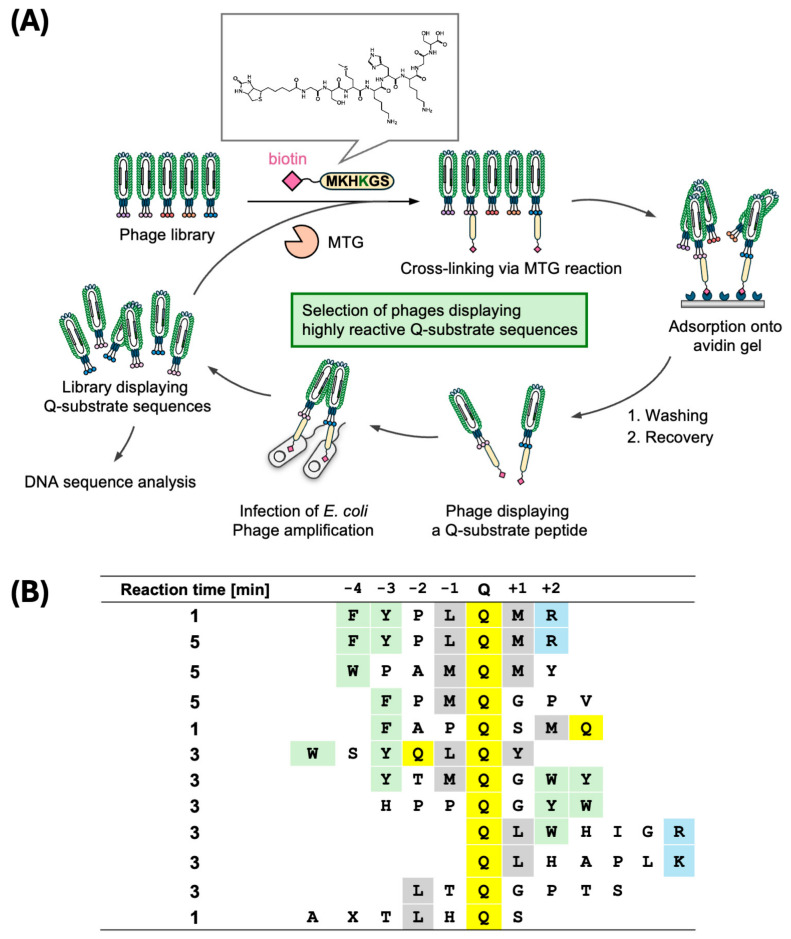
(**A**) Schematic illustration of the selection of preferred Gln-substrate peptide sequences for MTG using an M13 phage display random heptapeptide library with biotin-GSMKHKGS as bait. (**B**) Peptide sequences obtained from the screening of a random heptapeptide phage display library based on MTG-catalyzed cross-linking with biotin-GSMKHKGS as the Lys-acceptor substrate. Aromatic and hydrophobic residues are highlighted in green and gray, respectively. Gln residues and basic residues are indicated in yellow and blue, respectively.

First, we set up a phage display-based screening system to explore highly reactive Gln-donor substrates used for the MTG-catalyzed labeling of specific Lys residues in the protein of interest (Figure 1A). We followed the pioneering work by Sugimura et al. to screen preferred substrates of mammalian transglutaminases by using an M13 phage display system comprising 12 random amino acids [30]. They also reported screening for preferred Gln-donor substrates in the MTG-catalyzed cross-linking reaction using monodansyl cadaverine (a primary amine substrate) as a bait, identifying several unique substrates [21]. In this study, we used an M13 phage display system with random heptapeptides to search for a shorter Gln-donor substrate than those reported previously, using a highly reactive Lys-containing substrate (biotin-GSMKHKGS) as bait (Figure 1A), to explore an unmet Gln-donor substrate of MTG.

As a result, several Gln-containing peptide sequences were identified during screening (Figure 1B). The sequences lacking Gln residues are likely due to non-specific binding to the avidin used to capture biotinylated target sequences (Figure S1). Notably, many isolated Gln-containing sequences contain hydrophobic residues (Phe, Tyr, Trp, Leu, and Met) at the N-termini. This tendency is consistent with the structural features of the Gln-donor substrate recognition surface of MTG, which contains several hydrophobic amino acid residues, including Tyr, Val, Trp, Ile, and Phe. Therefore, hydrophobic residues located in the N-terminal region of the peptide are likely to facilitate substrate recognition through hydrophobic interactions with the enzyme active site. Among the isolated sequences, the peptide FYPLQMR appeared repeatedly in independent selections, suggesting that it represents a highly reactive Gln-donor substrate under the present screening conditions.

### 3.2. Design of an Azide-Labeled Gln-Donor Peptide for Chemoenzymatic Labeling of Fab Fragments

**Figure 2:**
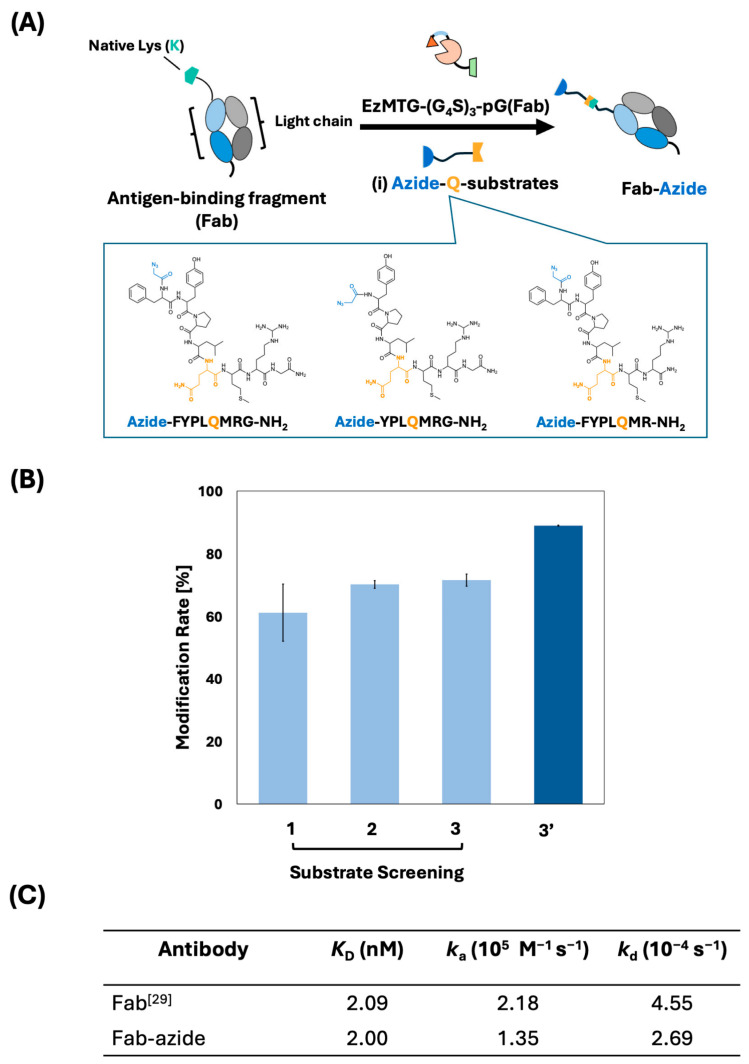
(**A**) Enzymatic labeling of Fab using three types of azido-functionalized Gln-donor substrates catalyzed by EzMTG-pG(Fab). (**B**) Left: RP-HPLC analysis of azide labeling to Fab via EzMTG-pG catalysis. Light blue columns represent the screening of the three types of azido-functionalized Gln-donor substrates. Substrate 1: azide-FYPLQMRG-NH_2_; Substrate 2: azide-YPLQMRG-NH_2_; Substrate 3: azide-FYPLQMR-NH_2_. The conjugation reaction was conducted under standard conditions: [Fab]/[MTG]/[Azido-Gln substrate] (μM) = 2.6/2.6/100; the dark blue column (3′) represents the modification rate of the conjugation reaction under increased concentration of substrate 3, where the concentration of the substrate was increased to 260 μM while the concentration of other components remained constant. The data are presented as the Mean ± SD of triplicate experiments (*N* = 3). (**C**) Evaluation of binding affinity of unmodified and azide-modified Fab (Fab-azide) towards HER2. *K*_D_: equilibrium dissociation constant; *k*_a_: association rate constant; *k*_d_: dissociation rate constant. Data were analyzed by global fitting using BLItz Pro (version 1.3. 1.3) software (ForteBio, Fremont, CA, USA).

Based on the findings presented above, we investigated the design of Gln-donor substrates suitable for click chemistry using the FYPLQMR sequence as the core sequence. Because the introduction of the highly hydrophobic DBCO group into relatively hydrophobic Gln-donor peptides could impair their aqueous solubility [29], we instead designed substrate peptides bearing a small azidoacetyl group at the N-terminus as a counterpart for a functional molecule with the DBCO moiety. We focused on the −4 (Phe) and +2 (Arg) positions relative to the Gln residue (Figure 1B). To evaluate how N-terminal hydrophobic residues and C-terminal positively charged residues affect substrate recognition, two additional peptides—azido-YPLQMRG-NH_2_ (lacking the N-terminal Phe) and azido-FYPLQMRG-NH_2_ (extended with Gly at the C-terminus)—were designed, in addition to the original sequence azido-FYPLQMR-NH_2_. Fab labeling experiments were then performed using these three Gln-donor substrate peptides designed for click chemistry (Figure 2A).

The enzymatic labeling of azide-functionalized Gln-donor substrates by EzMTG-pG(Fab) occurred selectively in the heavy chain (Figure S2), which is consistent with our previous reports [27,28]. However, due to their lower hydrophobicity compared to the DBCO moiety, azide modification of Fab had only a minor effect on its Fab hydrophobicity (Figure S2), as demonstrated by the overlapping peaks of the azide-modified and the unmodified heavy chains. Analysis of the modification rate from the deconvoluted peaks revealed that the azido-FYPLQMRG-NH_2_ was the least reactive (61.2 ± 9.2% modification ratio), while azido-YPLQMRG-NH_2_ and azido-FYPLQMR-NH_2_ showed comparable reactivity, with the latter resulting in a slightly higher modification ratio (70.2 ± 1.2% and 71.6 ± 2.0%, respectively) (Figure 2B). Despite the relatively lower labeling efficiency compared to our previous study with TAMRA-YPLQMRG-NH_2_ (>95% modification rate) [28], by increasing the concentration of the Gln-donor substrate, further improvement in modification rate was achieved to reach 89.0 ± 0.1% (Figure 2B).

**Figure 3:**
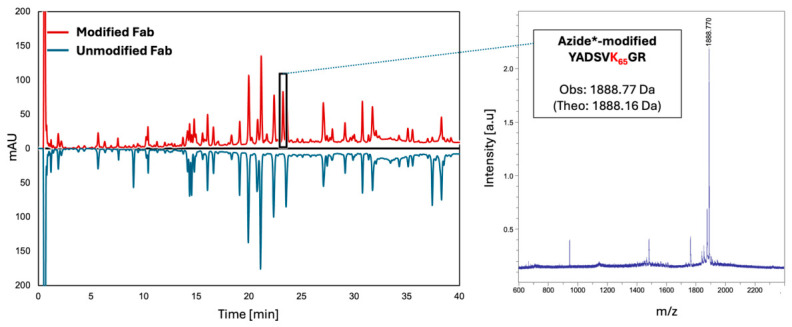
(**left**) Modification position analysis of azide-modified Fab. RP-HPLC chromatogram of digested azide-modified Fab and unmodified Fab. (**right**) The peak in the black square corresponds to the azide*-modified fragmented peptide containing the K65 residue. This peak was isolated, and its molecular weight was analyzed by MALDI-ToF-MS. Azide* corresponds to azide-modified YADSVK_65_GR, where the azide moiety was reduced to amino-modified YADSVK_65_GR during the preparation of the fragmented peptide.

After confirming the reactivity of the Gln-donor substrates, we evaluated the antigen-binding affinity of the Fab modified with azido-FYPLQMR-NH_2_ (Figure 2C and Figure S3). The azide-modified Fab retained antigen-binding ability relative to the unmodified Fab [29] (Figure 2C), indicating that the conjugation approach applied to the Fab did not negatively impact on the antigen-binding ability of the Fab. Analysis of the conjugation position showed that EzMTG-pG catalyzed the site-selective labeling of azido-FYPLQMR-NH_2_ to the K65 of the heavy-chain Fab (Figure 3). As observed in Figure 3, there appeared a new peak in azide-modified Fab that is absent in the unmodified Fab (Figure 3). This new peak corresponds to a peptide fragment containing K65, modified by one molecule of azido-FYPLQMR-NH_2_. The observed value was smaller than when the peptide fragment was attached to an intact azido-FYPLQMR-NH_2_ (azide-modified YADSVKGR = 1914.15 Da). The 25.99 Da discrepancy was due to the azide group (N_3_-peptide) being reduced to the amino group (NH_2_-peptide) during the reduction and alkylation process for peptide mapping analysis.

### 3.3. Preparing and Evaluation of a Fluorescent Fab Conjugate with a New Gln-Donor Substrate

**Figure 4:**
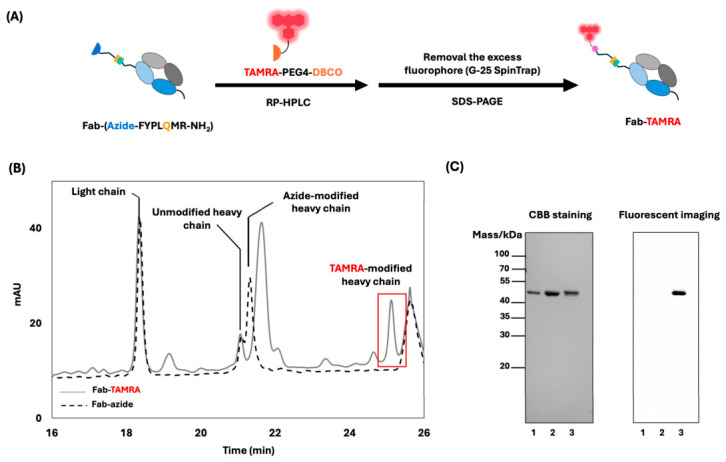
(**A**) Fluorescent labeling of the azide-modified Fab. (**B**) RP-HPLC chromatogram of click reaction mixture of azide-modified Fab with TAMRA-PEG4-DBCO. (**C**) SDS-PAGE analysis of TAMRA-modified Fab after click reaction under non-reducing condition. Lane 1: Fab; Lane 2: azide-modified Fab (Fab-azide); Lane 3: TAMRA-modified Fab (Fab-TAMRA).

Upon identifying azido-FYPLQMR-NH_2_ as the most reactive Gln-donor peptide, we used this substrate to construct a Fab-fluorescent conjugate, TAMRA-modified Fab (Fab-TAMRA), to further validate the azide labeling to Fab and demonstrate the functionality of the attached azide group (Figure 4A). Azide-functionalized Fab was obtained via EzMTG-pG(Fab) catalysis, which has previously demonstrated catalytic ability to modify K65 of the heavy-chain of Fab selectively [28,29]. The resulting azide-modified Fab was subsequently conjugated with TAMRA-PEG4-DBCO, as a model compound, via click chemistry. The RP-HPLC analysis showed that most of the azide-modified Fab had reacted with TAMRA-PEG4-DBCO as the minimum residual peak corresponding to the azide-modified heavy chain was detected (Figure 4B). SDS-PAGE analysis of the purified products showed that the chemoenzymatic modification of Fab had successfully occurred, resulting in Fab-TAMRA (lane 3, Figure 4C and Figure S4). These results demonstrated the potential of newly designed clickable Gln-donor substrates for antibody labeling studies.

## 4. Discussion

The FYPLQMR peptide, selected from the phage display library, demonstrated high reactivity as a Gln-donor substrate for MTG. Notably, this sequence partially resembles the representative MTG substrate peptide M42 (**Y**E**L**QRPYHSELP), previously identified by Sugimura et al., in which the residues at positions −3 (Tyr) and −1 (Leu) relative to the reactive Gln residue (highlighted in bold) are considered important for substrate recognition [21]. In the FYPLQMR peptide, the corresponding −3 position is also occupied by Tyr, suggesting that a similar recognition mode may operate in this peptide. By contrast, differences were observed at positions +1 and +2 relative to the Gln residue when compared with that of the M42 peptide. These differences may reflect the distinct screening conditions used in the present study. In our phage display selection, the cross-linking reaction was performed using the MTG-reactive Lys-containing peptide, MKHKGS [31], as the acceptor substrate. Because MTG-catalyzed cross-linking proceeds via the formation of an enzyme–Gln-donor intermediate (i.e., enzyme–substrate complex) that is subsequently attacked by a Lys-acceptor substrate, the microenvironment around the Gln residue may be influenced by the properties of the Lys-containing bait peptide. Thus, the sequence FYPLQMR likely represents a substrate optimized for cross-linking with the MKHKGS acceptor peptide used in the present screening system. Collectively, FYPLQMR was selected as a core sequence for further design of Gln-donor substrates.

In addition to FYPLQMR, two alternative Gln-donor substrate sequences, YPLQMRG and FYPLQMRG, were designed to evaluate the effect of an N-terminal Phe residue and a one-amino-acid extension at the C-terminus. The former sequence, YPLQMRG, previously demonstrated high reactivity as an acyl donor in EzMTG-pG catalysis when the model drug payload, TAMRA, was appended to the N-terminus [27,28]. Further modification of these Gln-donor substrates with chemical functionalities compatible with bioorthogonal strategies, such as click chemistry, may increase their modularity [32]. Click chemistry enables the specific conjugation of two complementary functionalities. Moreover, a wide variety of conjugation chemistries are currently available, and the incorporation of clickable handles does not necessarily require genetic engineering approaches.

In accordance with this, a chemoenzymatic approach specifically via SPAAC (strain-promoted azide-alkyne cycloaddition) has been explored herein. However, when YPLQMRG-NH_2_ was modified with a strained alkyne (DBCO) and conjugated to the Fab fragment, the resulting Fab-DBCO conjugate tended to aggregate [29]. This suggests that appending a hydrophobic clickable moiety to the Gln-donor substrate (i.e., DBCO-YPLQMRG-NH_2_) may compromise the aqueous solubility of the resulting Fab conjugate. In the case of antibody conjugates, the hydrophobicity of the linker-payload, including the attached clickable moiety, is one of the factors that can affect the propensity of the resulting conjugate to aggregate [33]. Considering that the chemoenzymatic strategy applied herein is a two-step process, maintaining the stability and solubility of the intermediate conjugate is essential. Therefore, attaching a less hydrophobic clickable handle, such as an azide group, to Gln-donor substrates could be beneficial in minimizing the aggregation propensity of the resulting proteinaceous conjugates. Indeed, the modification of an antibody with an azide linker has been widely reported [10,34,35,36], indicating that attachment of a small, less hydrophobic moiety is reliable and compatible in antibody engineering. Therefore, in this study we propose the first Gln-donor substrates with azide functionality and evaluate their labeling efficiency in MTG catalysis.

Comparison between azido-FYPLQMR-NH_2_ and azido-YPLQMRG-NH_2_ revealed that the N-terminal Phe residue contributes to the substrate recognition by MTG, although its effect is relatively modest. This observation is consistent with the importance of the −3 (Tyr) position for interaction with the MTG active site, which is enriched in hydrophobic amino acid residues. A similar trend has also been reported in previous studies, where the presence of N-terminal aromatic residue (at the −3 to −5 position relative to glutamine) constitutes the preferred substrate for MTG [21,23]. In contrast, despite sharing a similar sequence from position −3 to +3 with both of the preferred substrates, the azido-FYPLQMRG-NH_2_ demonstrated the lowest reactivity. This indicates that the use of a shorter peptide is more efficient, likely due to the steric hindrance imposed by the longer peptide when reacting with the target protein [37].

Herein, we report a phage-selected Gln-containing peptide scaffold as a potent substrate for MTG-mediated Lys-specific labeling to yield azide-functionalized Fab. This can then undergo further functionalization with a variety of chemical and biological entities by click chemistry. Examples range from the conjugation of drug molecules [32,38] to the formation of bispecific antibodies [4,39] using other antibody fragments, the attachment of proteins [40], and the incorporation of nanoparticles [41]. Such modularity would be difficult to achieve via direct enzymatic conjugation, as this would require genetic engineering to attach an enzyme recognition tag to the conjugation partner. The present results suggest the importance of the Lys-acceptor partner, as revealed in the phage selection using the reactive MKHKGS sequence. These results indicate that an optimal combination of Gln-donor and Lys-acceptor substrates is required to maximize the potential of MTG-catalyzed cross-linking reactions in biomolecular engineering applications.

## 5. Conclusions

In MTG-catalyzed cross-linking reactions, two types of substrates, a Gln-donor and a Lys-acceptor, participate in the enzymatic reaction. The Gln-donor substrate sequences identified by phage display using a highly reactive Lys-acceptor peptide as bait showed similarities in their N-terminal regions to those obtained using primary amine substrates. By contrast, differences were observed in the positions of Pro and Arg residues in the C-terminal region. The designed azide-functionalized Gln-donor substrate based on the identified sequence enabled efficient, site-selective Fab labeling via a proximity effect. This approach enables functional diversification via click chemistry. The present strategy provides a practical platform for preparing site-specifically functionalized antibodies and their fragments using EzMTG-pG fusion proteins.

## Data Availability

The original contributions presented in this study are provided in the article and Supplementary Material. Further inquiries can be directed to the corresponding author.

## Supplementary Materials

The following supporting information can be downloaded at: https://www.mdpi.com/article/10.3390/antib15040056/s1.

## Abbreviations

The following abbreviations are used in this manuscript:

Fab	Antigen-binding fragment of antibody
DBCO	Dibenzocyclooctyne
MTG	Microbial transglutaminase
EzMTG-pG	Engineered zymogen of MTG fused with protein G
HER2	Human epidermal growth factor receptor 2
SPAAC	Strain-promoted azide-alkyne cycloaddition
