# Supplementary material for: Phage-Selected Clickable Gln-Donor Peptide for Lys-Selective Fab Labeling Using Engineered Microbial Transglutaminase
Source: Antibodies (Basel). 2026 Jun 26;15(4):56. doi: 10.3390/antib15040056 (PMC13398322; doi:10.3390/antib15040056)
Supplement: Supplementary file 1 [file antibodies-15-00056-s001.zip › antibodies-4318767-supplementary.pdf]

Supplementary Materials

Table of contents

1.	Supplementary results	S1
	1-1 The sequences lacking Gln residues identified from the screening	S2
	1-2 Conjugation of Fab with three types of azide-modified Gln-donor substrates under EzMTG- pG(Fab) catalysis	S3
	1-3 Representative sensorgram of the binding affinity evaluation of the azide-modified Fab (Fab-azide) to HER2.	S4
	1-4 Raw images of SDS-PAGE analysis of TAMRA-modified Fab for Fig. 4C.	S5

1. Supplementary results

1-1. The sequences lacking Gln residues identified from the screening

Numbers of clones	Selected sequence (w/o Q)							
22	W	P	G	L	L	A	G	
2	W	P	G	I	L	A	H	
1	Y	P	G	A	G	D	S	
3	W	P	G	I	K	A	H	
1	A	V	G	V	R	L	A	
1	A	P	R	H	A	Y	A	
1	G	P	F	S	A	S	P	
1	S	I	V	I	K	S	T	
1		L	A	V	S	H	T	E
1	S	M	M	P	V	P	P	
1	T	M	L	S	A	P	L	
1	V	D	P	W	R	V	R	
1	X	X	X	Y	N	X	X	

Fig. S1. The peptide sequences lacking Gln residue obtained from the screening of random heptapeptide library based on MTG-catalyzed crosslinking with biotin-GSMKHKGS. Aromatic and hydrophobic residues are shown in green and gray, respectively. Pro and Gly are shown in orange and blue, respectively. Basic residues are shown in blue.

1-2. Conjugation of Fab with three types of azide-modified Gln-donor substrates under EzMTG-pG(Fab) catalysis

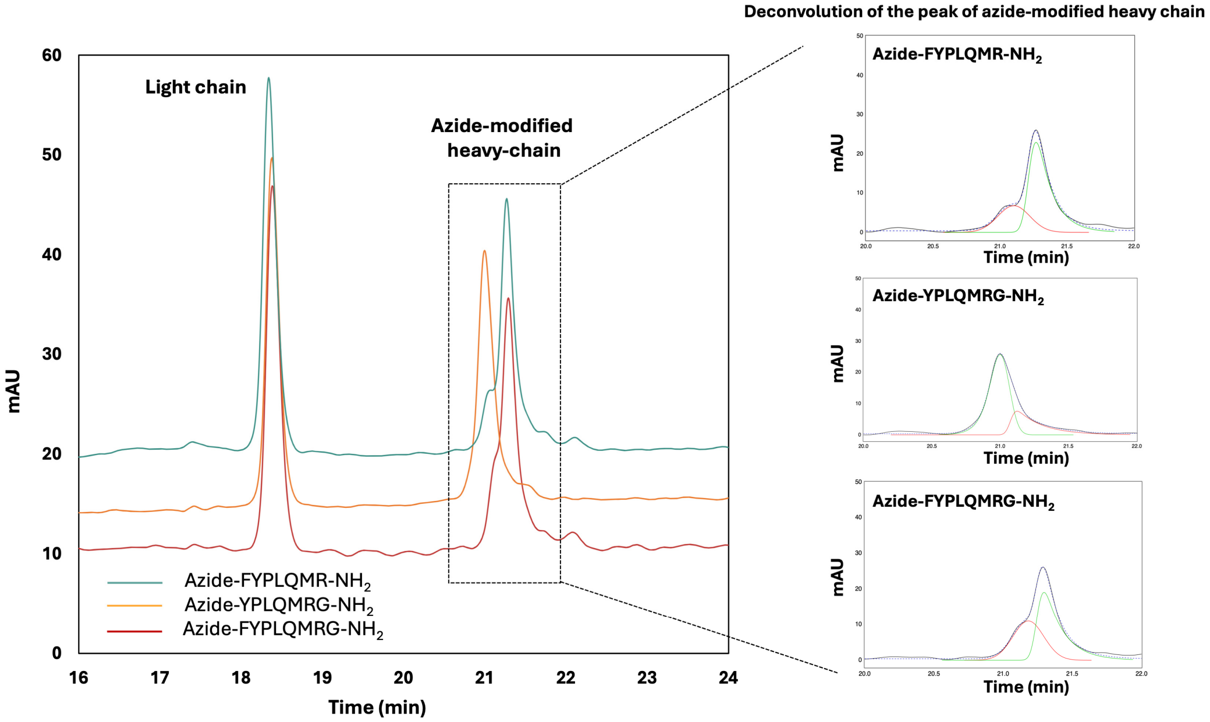

Fig. S2. Conjugation of Fab with three types of azide-modified Gln-donor substrates under EzMTG-pG catalysis. Left: RP-HPLC chromatogram of azide-modified Fab. Right: Deconvoluted peak of azide-modified heavy-chains. Black line: azide-modified heavy-chain peak (experimental data); Blue dashed line: the cumulative fit of the experimental data by multipeak deconvolution model; Green line: Fitted peak of azide-modified heavy chain; Red line: Fitted peak of unmodified heavy chain.

1-3. Representative sensorgram of the binding affinity evaluation of the azide-modified Fab (Fab-azide) to HER2.

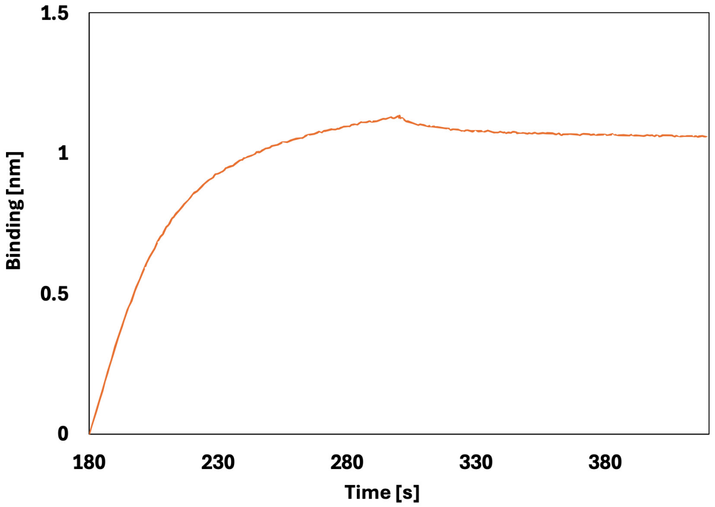

Fig. S3. Representative sensorgram of the binding affinity evaluation of the azide-modified Fab (Fab-azide) (250 nM) to HER2 by bio-layer interferometry (BLI) analysis.

1-4. Raw images of SDS-PAGE analysis of TAMRA-modified Fab for Fig. 4C.

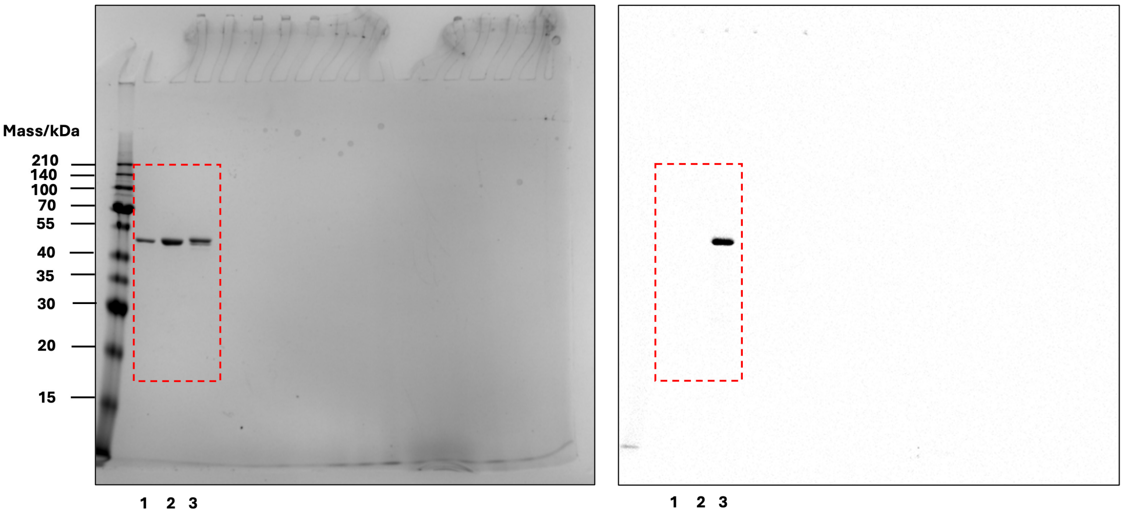

Fig. S4. Raw images of SDS-PAGE analysis of TAMRA-modified Fab under non-reducing condition for Fig. 4C (rectangular boxes with red dashed line). Left: CBB-staining; Right: Fluorescence imaging. Lane 1: Fab; Lane 2: Azide-modified Fab (Fab-azide); Lane 3: TAMRA-modified Fab (Fab-TAMRA).
